# Risk of hepatic and extrahepatic cancer in NAFLD: A population‐based cohort study

**DOI:** 10.1111/liv.15195

**Published:** 2022-02-24

**Authors:** Karl Björkström, Linnea Widman, Hannes Hagström

**Affiliations:** ^1^ Department of Medicine Huddinge, Karolinska Institutet Stockholm Sweden; ^2^ Division of Biostatistics Institute of Environmental Medicine, Karolinska Institutet Stockholm Sweden; ^3^ Division of Hepatology, Department of Upper Gastrointestinal Diseases Karolinska University Hospital Stockholm Sweden; ^4^ Clinical Epidemiology Unit, Department of Medicine Solna, Karolinska Institutet Stockholm Sweden

**Keywords:** cancer, diabetes, hepatocellular carcinoma, NAFLD

## Abstract

**Background and Aims:**

Individuals with non‐alcoholic fatty liver disease (NAFLD) may be at greater risk of cancer. This study aimed to investigate the risk of hepatic and extrahepatic cancer compared to the general population in a population‐based cohort of patients with NAFLD.

**Methods:**

We used the Swedish National Patient Registry from 1987 to 2016 to identify patients with a NAFLD diagnosis and no prior cancer. All patients with NAFLD were compared to up to 10 controls matched for age, sex and living location. The primary outcome was the first occurrence of any cancer as ascertained from national registries. As secondary outcomes, we analysed the risk of pre‐specified cancer subtypes. Cox regression models, adjusted for baseline diabetes, hypertension, hyperlipidaemia and chronic obstructive pulmonary disease were applied.

**Results:**

We identified 8415 patients with NAFLD. Over a median follow‐up of 6.0 years (IQR 2.5–11.2 years), an increased risk for any cancer was found in patients with NAFLD compared to controls (9.7 vs. 8.6 cases per 1000 person‐years): hazard ratio (HR) = 1.22 (95% confidence interval, CI = 1.12–1.33). The risk for hepatocellular carcinoma (HCC) was particularly high (adjusted HR, aHR = 12.18, 95% CI = 7.15–20.79). The risk for some other cancer subtypes increased (colorectal [aHR 1.38], kidney [aHR 2.12], bladder [aHR 2.51] and uterine [aHR 1.78]), but was low in absolute terms.

**Conclusion:**

In this population‐based cohort, NAFLD was associated with an increased risk of developing cancer (especially HCC). The absolute risk for other forms of cancer was generally comparable to the control population.

## Lay summary

- Patients with NAFLD have an increased risk of liver cancer. The risk of other cancers in patients with NAFLD is unclear.
- We examined the risk of cancer in general, as well as specific types of cancer in Swedish patients with NAFLD.
- NAFLD was associated with an increased risk of liver cancer, independent of liver cirrhosis. A slightly increased risk of other types of common cancers was also observed in patients with NAFLD compared to individuals without NAFLD.

**Abbreviations**
aHR: adjusted hazard ratio
COPD: chronic obstructive pulmonary disease
HCC: hepatocellular carcinoma
HR: hazard ratio
ICD: international classification of disease
IR: incidence rate,
NAFLD: non‐alcoholic fatty liver disease
NPR: national patient registry
PPV: positive predictive value
SCR: Swedish cancer registry
sHR: subdistribution hazard ratio
T2D: type 2 diabetes

## 1 INTRODUCTION

In recent decades, the increasing prevalence of obesity and type 2 diabetes (T2D) has been accompanied by an increase in non‐alcoholic fatty liver disease (NAFLD). The global prevalence of NAFLD is estimated to be about 24%.^1^, ^2^, ^3^ Increasing attention has focused on the hepatic (e.g. cirrhosis and hepatocellular carcinoma (HCC)) and extrahepatic (e.g. T2D, cardiovascular disease and extrahepatic cancers) complications of NAFLD.^4^, ^5^ An increased risk of HCC in patients with NAFLD has been repeatedly reported.^6^, ^7^, ^8^, ^9^, ^10^ It was also recently noted that cancers are a significant contributor to mortality in patients with biopsy‐proven NAFLD.^11^

However, the absolute risk of HCC in patients with NAFLD without cirrhosis has been reported to be low.^10^ Mantovani et al. recently published a meta‐analyses of 10 cohorts of over 180 000 individuals, mostly originating from Asia, and reported NAFLD to be associated with an increased risk of several extrahepatic cancers compared to reference individuals free of NAFLD.^12^ In a study of a Chinese cohort of males with NAFLD without cirrhosis, an increased risk of any cancer was found compared to controls without NAFLD.^13^ Allen et al. reported an increased risk of cancer in patients diagnosed with NAFLD compared to controls without NAFLD. In this same study, no increased risk of cancer was seen in obese patients without a diagnosis of NAFLD.^8^ Other studies have shown an increased risk of colorectal cancer in males with NAFLD, but not females with NAFLD.^7^, ^8^, ^14^ However, in a meta‐analysis from 2018, an increased risk of colorectal cancer was found in patients with NAFLD independent of sex.^15^

The risk of breast cancer has been shown to increase in NAFLD.^7^, ^8^, ^16^ In contrast, some studies have found an increased risk of breast cancer only in non‐obese^17^ or post‐menopausal^18^ women with NAFLD. For other cancers, such as lung, oesophageal, prostate and bladder cancer, results have been conflicting and lack replication.^7^, ^8^, ^13^, ^19^, ^20^, ^21^

The results from previous studies have been inconclusive, which could be as a result of different methods with varying sensitivity and specificity for diagnosing NAFLD (e.g. non‐invasive scores based on blood samples and body mass index,^10^, ^18^, ^21^ lack of adjusting for important confounders^6^ and studying selected, non‐population‐based cohorts).^7^, ^10^, ^14^, ^16^, ^17^, ^19^ A need for further studies on the association between NAFLD and cancer has been suggested.^4^

We aimed to investigate the risk of cancer in a population‐based cohort of patients diagnosed with NAFLD, with and without cirrhosis, compared to the general population.

## 2 MATERIALS AND METHODS

### 2.1 Study population

We used the Swedish National Patient Registry (NPR) to identify all patients diagnosed with NAFLD in Sweden from 1 January 1987 to 31 December 2016. The NPR contains data on all patients discharged from hospitals in Sweden, and from 2001 the registry also contains data on all specialized care outpatient visits. The NPR’s positive predictive value (PPV) for most chronic diseases ranges between 85% and 95%.^22^ The PPV is 91% for HCC patients with established liver disease.^23^ Each patient with NAFLD was matched on sex, age, county of residence and calendar year of diagnosis with up to 10 controls free of NAFLD obtained from Statistics Sweden. The International Classification of Disease (ICD) codes used to identify patients with NAFLD were 571.8 in ICD‐9 and K75.8 or K76.0 in ICD‐10. We defined the presence of cirrhosis using the ICD codes 571.5 in ICD‐9 and K74.6 in ICD‐10.

**FIGURE 1:**
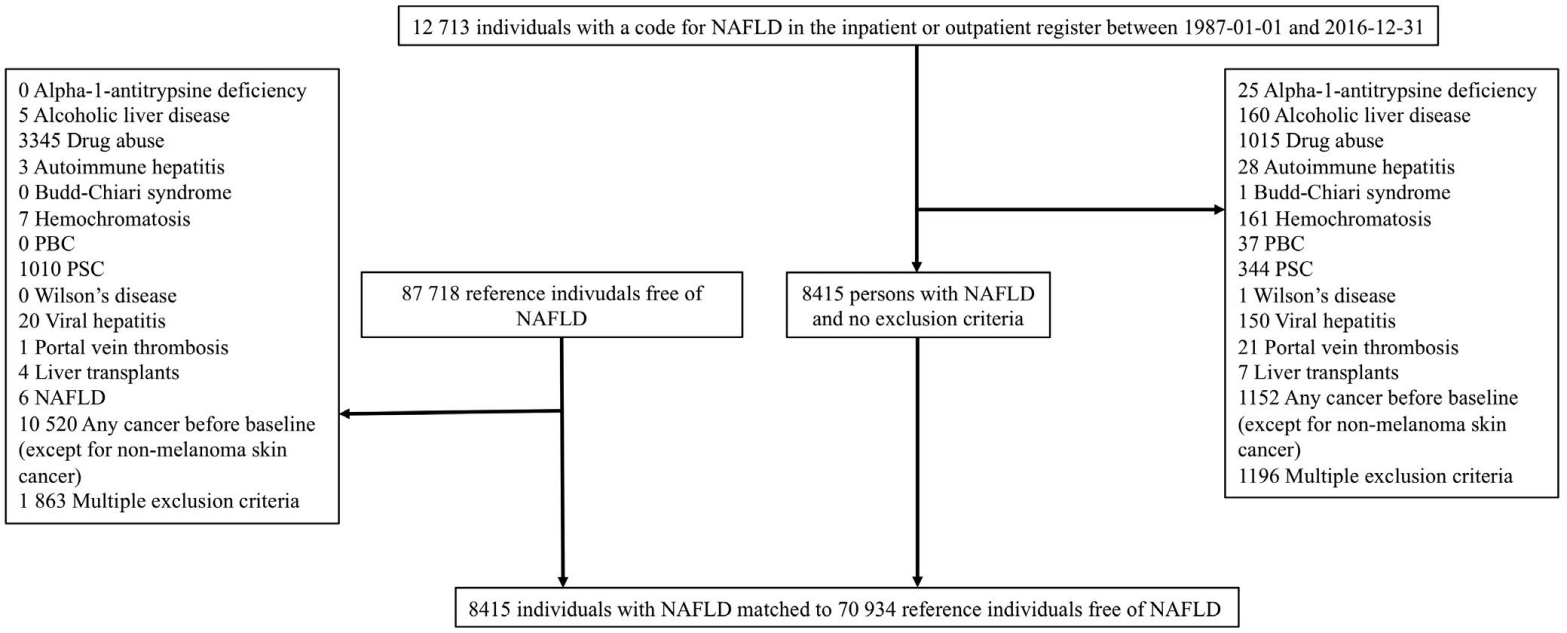
Flow chart of study participants

We excluded patients with NAFLD and controls with any of the following: liver diseases other than NAFLD, a history of drug or alcohol abuse, previous liver transplant and any cancer except for non‐melanoma skin cancer before baseline (Figure 1). We censored patients diagnosed during follow‐up for another liver disease or a diagnosis of alcohol or drug abuse. Study participants were further censored at emigration from Sweden, death or liver transplantation, whichever is applicable (*eTable* 1 presents specific ICD codes).

### 2.2 Outcomes

The primary outcome was time to the first diagnosis of any type of cancer (ICD‐7: 140–207 except 191), except non‐melanoma skin cancer in the Swedish Cancer Registry (SCR). The SCR has data on approximately 96% of all cancer diagnoses in Sweden.^24^ Secondary outcomes were time to the first diagnosis of the following cancers in the SCR: HCC, colorectal, gastric, kidney, bladder, cervical, ovarian, uterine, breast, lung, oesophageal and prostate cancer (see *eTable 1* for ICD codes). Diagnoses of cancer were not deemed outcomes if they occurred earlier than 1 year after baseline. When analysing secondary outcomes, we were interested in specific cancer diagnoses even if they did not occur as a first cancer during follow‐up. Hence, if a patient had a first diagnosis of cancer that occurred earlier than 1 year after baseline, and then the second diagnosis of another kind of cancer specified as secondary outcome later than 1 year after baseline, the second diagnosis was counted as the outcome in the analysis of secondary outcomes.

To investigate whether the risk of cancer could be influenced by differences between patients with NAFLD and controls in non‐cancer mortality, we used data from the Causes of Death Registry, which contains information on the cause of death for all citizens in Sweden.^25^ We defined causes of death as either cancer related or non‐cancer related. Cancer‐related death was defined as having an ICD code of any cancer, except for a non‐melanoma skin cancer diagnosis as a primary or secondary cause of death.

### 2.3 Covariates

We included diabetes, hypertension, hyperlipidaemia and chronic obstructive pulmonary disease (COPD) as covariates in the regression models. Diabetes, hypertension and hyperlipidaemia were included as markers of metabolic health, which is related both to NAFLD and risk of cancer. The used registers do not contain more detailed data on possible confounders, such as waist circumference, body mass index plasma glucose or blood lipid profiles. Because of the lack of direct data on smoking, we included COPD as a proxy for smoking. All covariates were defined as the corresponding ICD codes in the NPR, and both primary and secondary codes were used to identify covariates (*eTable* 1 lists the specific ICD codes).

### 2.4 Statistical analysis

Differences between baseline variables of patients with NAFLD with and without cirrhosis at baseline were calculated using Fischer’s exact test for categorical variables and Wilcoxon rank‐sum test for continuous variables. We estimated incidence rates (IRs) per 1000 person‐years for primary and secondary outcomes as the total number of outcomes divided by person‐years of follow‐up. Univariate and multivariable (adjusted for diabetes, hypertension, hyperlipidaemia and COPD) Cox regression models were used to estimate the association between NAFLD and the primary and secondary outcomes. We chose to use the same adjustment factors for all cancer subtypes to improve model comparability between these outcomes.

### 2.5 Sensitivity analyses

First, we investigated the impact of cirrhosis on cancer risk, comparing patients without cirrhosis to their respective controls.

Second, given that the risk of any cancer might be positively influenced by an unbalanced risk for HCC in patients with NAFLD, one sensitivity analysis excluded all individuals in which HCC was the first diagnosed cancer.

Third, because the risk of HCC might be attributed to cirrhosis not diagnosed at baseline but instead detected during follow‐up, one analysis excluded any patient who developed cirrhosis during follow‐up.

Fourth, in an adjunct analysis, we applied a competing risk regression in which non‐cancer death was the competing risk and adjusted for the same covariates as the adjusted Cox model. This analysis was done because the risk of death might be higher in the NAFLD population, possibly inflating the estimates for cancer risk.

Finally, we examined the risk of all cancers and secondary outcomes in males and females separately.

## 3 RESULTS

Some 8415 patients with NAFLD and 70 934 controls were included (Figure 1). At baseline, study participants had a median age of 53 years and 56% were men. In patients with NAFLD, baseline comorbidities were more common than in controls (Table 1). A majority (66.1%) of patients with NAFLD were diagnosed between 2010 and 2016. Of patients with NAFLD, 183 (2.2%) had cirrhosis at baseline. Detailed baseline characteristics are presented in Table 1. The median follow‐up was 6.0 years (IQR 2.5–11.2 years).

**TABLE 1:** Baseline characteristics for the study population. Patients with cirrhosis at baseline had a concurrent or previous diagnosis of cirrhosis before receiving a diagnosis of NAFLD. Differences between baseline variables of patients with NAFLD with and without cirrhosis were calculated using Wilcoxon rank‐sum test for continuous variables and Fischer’s exact test for categorical variables

	Entire cohort			Patients with NAFLD		
	Patients with NAFLD, *n* = 8415	Reference individuals, *n* = 70 934		Cirrhosis at baseline, *n* = 183	No cirrhosis at baseline, *n* = 8232	
Age, years, median (IQR)	54 (23)	53 (23)	*p* < .01	62 (12)	54 (24)	*p* < .01
Females (%)	3844 (45.7)	31 222 (44.0)	*p* < .01	81 (44.3)	4490 (54.5)	*p* < .01
Baseline comorbidities						
Cirrhosis, *n* (%)	183 (2.2)	0 (0.0)	—	183 (100)	—	—
Diabetes, *n* (%)	1607 (19.1)	2407 (3.4)	*p* < .01	105 (57.4)	1502 (18.2)	*p* < .01
COPD, *n* (%)	280 (3.3)	614 (0.9)	*p* < .01	12 (6.6)	268 (3.3)	*p* = .02
Hypertension, *n* (%)	2184 (25.6)	5218 (7.4)	*p* < .01	85 (46.4)	2086 (25.3)	*p* < .01
Hyperlipidaemia, *n* (%)	937 (11.1)	1804 (2.5)	*p* < .01	35 (19.1)	902 (11.0)	*p* < .01
Liver biopsy within 1 year before baseline, *n* (%)	481 (5.7)	2 (0.0)	*p* < .01	24 (13.1)	457 (5.6)	*p* < .01
Year of diagnosis						
1987–1989, *n* (%)	160 (1.9)	—	—	3 (1.6)	157 (1.9)	*p* = 1.00
1990–1999, *n* (%)	397 (4.7)	—	—	4 (2.2)	393 (4.8)	*p* = .11
2000–2009, *n* (%)	2298 (27.3)	—	—	31 (16.9)	2267 (27.5)	*p* < .01
2010–2016, *n* (%)	5560 (66.1)	—	—	145 (79.2)	5415 (65.8)	*p* < .01

We identified 527 (6.3%) cancer cases in patients with NAFLD and 4716 (6.6%) in controls. The IR per 1000 person‐years was 9.7 (95% CI = 8.9–10.6) in NAFLD compared to 8.6 (95% CI = 8.3–8.9) in controls, corresponding to an adjusted HR (aHR) of 1.22 (95% CI = 1.11–1.33). For HCC, we found 47 (0.5%) cases in patients with NAFLD and 34 (0.05%) in controls. The aHR for HCC in patients with NAFLD compared to controls was 12.2 (95% CI = 7.1–20.8). Results for all secondary outcomes are summarized in Table 2.

**TABLE 2:** Total number of cancer diagnoses, incidence rates per 1000 person‐years and crude and adjusted hazard ratios for incident cancers in patients diagnosed with NAFLD in Sweden between 1987 and 2016 compared to age‐, sex‐ and living location‐matched reference individuals

Type of cancer	Incident cases		Incidence rate (95% CI) per‐1000 PY		HR (95% CI)	aHR (95% CI)
	NAFLD (*n*, %)	Reference individuals (*n*, %)	NAFLD	Reference individuals		
All cancers	527 (6.3)	4716 (6.6)	9.7 (8.9–10.6)	8.6 (8.3–8.9)	1.22 (1.12–1.32) (*p* < .01)	1.22 (1.12–1.33) (*p* < .01)
All cancers except if first cancer was HCC	483 (5.7)	4688 (6.6)	9.0 (8.2–9.8)	8.6 (8.3–8.8)	1.14 (1.04–1.24) (*p* < .01)	1.15 (1.05–1.26) (*p* < .01)
HCC	47 (0.6)	34 (0.0)	0.8 (0.6–1.1)	0.1 (0.0–0.1)	15.50 (9.92–24.21) (*p* < .01)	12.18 (7.15–20.79) (*p* < .01)
Colon and rectum	77 (0.9)	649 (0.9)	1.4 (1.1–1.7)	1.1 (1.1–1.2)	1.35 (1.08–1.69) (*p* = .01)	1.38 (1.09–1.75) (*p* < .01)
Stomach	10 (0.1)	77 (0.1)	0.2 (0.1–0.3)	0.1 (0.1–0.2)	1.52 (0.80–2.88) (*p* = .21)	1.32 (0.66–2.64) (*p* = .44)
Kidney	21 (0.2)	110 (0.2)	0.4 (0.2–0.6)	0.2 (0.2–0.2)	2.27 (1.46–3.52) (*p* < .01)	2.12 (1.34–3.36) (*p* < .01)
Bladder	37 (0.4)	199 (0.3)	0.7 (0.5–0.9)	0.4 (0.3–0.4)	2.37 (1.70–3.28) (*p* < .01)	2.51 (1.79–3.53) (*p* < .01)
Cervix	12 (0.1)	148 (0.2)	0.2 (0.1–0.4)	0.3 (0.2–0.3)	0.80 (0.46–1.41) (*p* = .45)	0.98 (0.56–1.70) (*p* = .94)
Ovary	8 (0.1)	85 (0.1)	0.1 (0.1–0.3)	0.1 (0.1–0.2)	0.98 (0.49–1.96) (*p* = .96)	0.93 (0.45–1.96) (*p* = .86)
Uterus	25 (0.0)	134 (0.2)	0.4 (0.3–0.7)	0.2 (0.2–0.3)	1.80 (1.21–2.68) (*p* < .01)	1.78 (1.18–2.68) (*p* < .01)
Breast	69 (0.8)	624 (0.9)	1.2 (1.0–1.6)	1.1 (1.0–1.2)	1.14 (0.90–1.44) (*p* = .27)	1.12 (0.87–1.45) (*p* = .37)
Lung	36 (0.4)	388 (0.5)	0.6 (0.5–0.9)	0.7 (0.6–0.7)	0.98 (0.71–1.34) (*p* = .90)	0.98 (0.71–1.36) (*p* = .93)
Oesophagus	2 (0.0)	42 (0.1)	0.0 (0.0–0.1)	0.1 (0.1–0.1)	0.45 (0.1–1.8) (*p* = .26)	0.52 (0.12–2.24) (*p* = .38)
Prostate	74 (0.9)	1024 (1.4)	1.3 (1.0–1.7)	1.8 (1.7–1.9)	0.81 (0.65–1.01) (*p* = .07)	0.88 (0.70–1.11) (*p* = .29)

*Note*. aHR = Adjusted for diabetes, hypertension, hyperlipidaemia and COPD.

Abbreviations: aHR, adjusted hazard ratio; CI, confidence interval; COPD, chronic obstructive pulmonary disease; NAFLD, non‐alcoholic fatty liver disease; PY, person‐years.

The analysis of cancer risk in patients with NAFLD without cirrhosis at baseline yielded similar results to the main analysis (Table S1). Because only nine cancer cases occurred in patients with NAFLD and cirrhosis at baseline, no analysis was performed separately for this group.

In the analysis, in which individuals whose first diagnosis of cancer was HCC were excluded, we found 483 (5.7%) cases of any non‐HCC cancer in patients with NAFLD and 4688 (6.6%) in controls. The IR per 1000 person‐years was 9.0 (95% CI = 8.2–9.8) in patients with NAFLD and 8.6 (8.3–8.8) in controls, with an aHR of 1.15 (95% CI = 1.05–1.26).

The sensitivity analysis of the risk of HCC in patients with NAFLD but without baseline cirrhosis (patients with NAFLD who developed cirrhosis during follow‐up [*n* = 10] were excluded) yielded similar results to the main analysis (aHR = 8.33, 95% CI = 4.70–14.75).

In the sex‐stratified analysis, we found 265 cases (5.8%) of any cancer in males with NAFLD compared to 2568 cases (6.5%) in controls. The IR per 1000 person‐years was 8.5 (95% CI = 7.6.0–9.6) in men with NAFLD compared to 8.1 (95% CI = 7.8–8.5) in controls. The aHR was 1.18 (95% CI = 1.04–1.34). In women with NAFLD, we found 262 cases (6.8%) of cancer compared to 2148 cases (6.9%) in controls. The IR per 1000 person‐years was 11.2 (95% CI = 10.0–12.7) in women with NAFLD compared to 9.3 (95% CI = 8.9–9.7) in controls. The aHR was 1.26 (95% CI = 1.11–1.43). An association between NAFLD and the risk of HCC, kidney and bladder cancer was found in males and females. In contrast, an association between NAFLD and risk of colorectal cancer was found in males (aHR = 1.54, 95% CI = 1.13–2.09) but not in females (aHR = 1.21, 95% CI = 0.84–1.73). No association was observed between NAFLD and the risk of other secondary outcomes in males or females. Table 3 presents data on sex‐stratified cancer risk estimates.

**TABLE 3:** Total number of cancer diagnoses, incidence rates per 1000 person‐years and crude and adjusted hazard ratios for incident cancers in men and women with a diagnosis of NAFLD in Sweden between 1987 and 2016 compared to age‐, sex‐ and living location‐matched reference individuals

Type of cancer	Incident cases		Incidence rate (95% CI) per‐1000 PY		HR (95% CI)	aHR (95% CI)
	NAFLD (*n*, %)	Reference individuals (*n*, %)	NAFLD	Reference individuals		
Women	*n* = 3844	*n* = 31 222				
All cancers	262 (6.8)	2148 (6.9)	11.2 (10.0–12.7)	9.3 (8.9–9.7)	1.27 (1.13–1.43) (*p* < .01)	1.26 (1.11–1.43) (*p* < .01)
HCC	17 (0.4)	9 (0.0)	0.7 (0.4–1.0)	0.0 (0.0–0.1)	16.37 (7.61–35.23) (*p* < .01)	8.74 (3.39–22.55) (*p* < .01)
Colon and rectum	32 (0.8)	297 (1.0)	1.3 (0.9–1.8)	1.2 (1.1–1.4)	1.18 (0.83–1.67) (*p* = .37)	1.21 (0.84–1.73) (*p* = .31)
Kidney	9 (0.2)	32 (0.1)	0.4 (0.2–0.7)	0.1 (0.1–0.2)	3.27 (1.67–6.41) (*p* < .01)	3.02 (1.50–6.05) (*p* < .01)
Bladder	11 (0.3)	51 (0.2)	0.4 (0.2–0.8)	0.2 (0.2–0.3)	2.61 (1.41–4.84) (*p* < .01)	2.81 (1.49–5.31) (*p* < .01)
Men	*n* = 4571	*n* = 39 712				
All cancers	265 (5.8)	2568 (6.5)	8.5 (7.6–9.6)	8.1 (7.8–8.5)	1.16 (1.03–1.31) (*p* = .01)	1.18 (1.04–1.34) (*p* < .01)
HCC	30 (0.7)	25 (0.1)	0.9 (0.6–1.3)	0.1 (0.0–0.1)	15.06 (8.70–26.08) (*p* < .01)	14.54 (7.35–28.74) (*p* < .01)
Colon and rectum	45 (1.0)	352 (0.9)	1.4 (1.1–1.9)	1.1 (1.0–1.2)	1.51 (1.12–2.04) (*p* < .01)	1.54 (1.13–2.08) (*p* < .01)
Kidney	12 (0.3)	78 (0.2)	0.4 (0.2–0.7)	0.2 (0.2–0.3)	1.84 (1.03–3.30) (*p* = .04)	1.87 (1.02–3.43) (*p* = .04)
Bladder	26 (0.6)	148 (0.4)	0.8 (0.6–1.2)	0.5 (0.4–0.5)	2.27 (1.54–3.35) (*p* < .01)	2.43 (1.62–3.65) (*p* < .01)

*Note*. aHR = Adjusted for diabetes, hypertension, hyperlipidaemia and COPD.

Abbreviations: aHR, adjusted hazard ratio; CI, confidence interval; COPD, chronic obstructive pulmonary disease; NAFLD, non‐alcoholic fatty liver disease; PY, person‐years.

**FIGURE 2:**
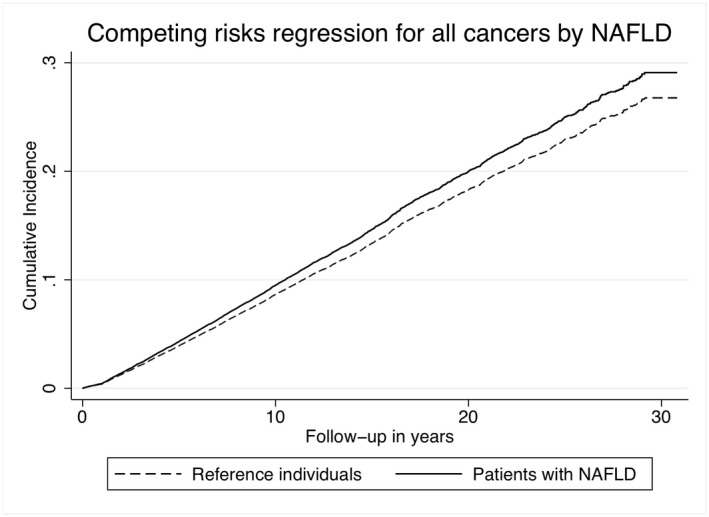
Cumulative incidence of all cancers in patients with NAFLD and reference individuals. Adjusted for baseline diabetes, hypertension, hyperlipidaemia and chronic obstructive pulmonary disease

**FIGURE 3:**
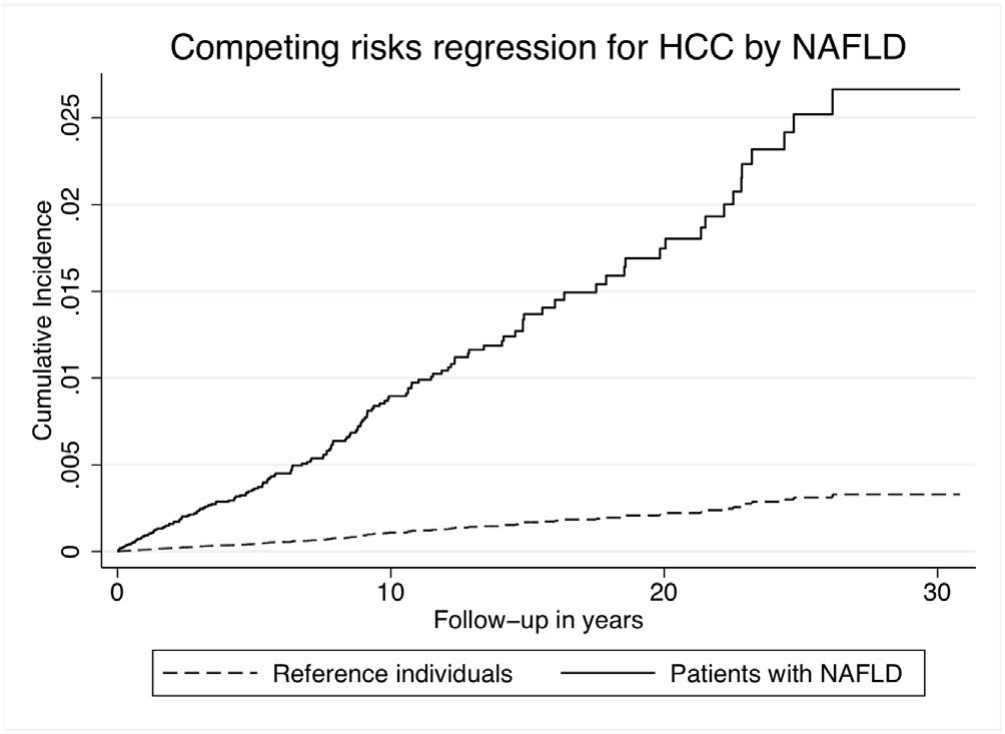
Cumulative incidence of HCC in patients with NAFLD and reference individuals. Adjusted for baseline diabetes, hypertension, hyperlipidaemia and chronic obstructive pulmonary disease

In the competing risk regression, NAFLD was independently associated with an increased risk of any cancer (subdistribution HR [sHR] = 1.10, 95% CI = 1.02–1.20, *p* = .02). Figure 2 shows the cumulative incidence for any cancer in NAFLD and controls. Moreover, NAFLD was independently associated (sHR = 8.16, 95% CI 5.68–11.75, *p* < .01) with the risk of HCC and death from HCC, accounting for death from other causes as the competing risk. Table S2 shows unadjusted and adjusted sHRs. Figure 3 displays the cumulative incidence for HCC in patients with NAFLD and controls. In Table 4, cumulative incidence for all cancer and death by any cancer at 5, 10 and 15 years of follow‐up is shown.

**TABLE 4:** Cumulative incidence for all cancers or death by any cancer at 5, 10 and 15 years of follow‐up for patients with NAFLD and matched reference individuals

	NAFLD (95% CI)	Reference individuals (95% CI)
All cancers		
5‐year follow‐up	3.6% (3.1–4.1)	3.3% (3.2–3.5)
10‐year follow‐up	8.5% (7.7–9.4)	7.7% (7.4–8.0)
15‐year follow‐up	12.9% (11.7–14.2)	12.1% (11.7–12.5)
Death by any cancer		
5‐year follow‐up	1.7% (1.4–2.1)	0.5% (0.5–0.6)
10‐year follow‐up	2.4% (2.0–2.8)	0.9% (0.8–1.0)
15‐year follow‐up	2.9% (2.4–3.4)	1.2% (1.1–1.4)

Abbreviations: CI, confidence interval; NAFLD, non‐alcoholic fatty liver disease.

## 4 DISCUSSION

In this population‐based cohort study of over 8000 patients with NAFLD and matched controls, we found an association between NAFLD and an increased risk of cancer. The increased risk of cancer was mainly attributed to HCC, but an increased risk for other cancers (colorectal cancer in men, bladder, kidney and uterine cancer) was also observed. The absolute increase in risk for non‐HCC cancers was low compared to matched controls. While the proportion of patients with NAFLD who developed cancer during follow‐up was similar in controls (6.3% vs. 6.6%), the incidence rate of cancer was higher in patients with NAFLD (9.7 per 1000 PYs vs. 8.6 in controls).

Most patients with NAFLD in our cohort (66.1%) were diagnosed with NAFLD during the last 7 years of the study period (2010–2016), which could be as a result of a true increase in NAFLD incidence. However, it could also be because of an increased awareness of NAFLD and improved disease awareness among healthcare professionals. We observed similar risk estimates for our outcomes after adjusting for relevant covariates (diabetes, hypertension, hyperlipidaemia and COPD), indicating that the observed associations between NAFLD and cancer are independent of these risk factors. The estimates obtained from a competing risk regression were also significant, suggesting that the observed increased risk for cancer is not solely as a result of higher mortality in patients with NAFLD compared to controls.

Kim et al. investigated the risk of cancer in 25 947 individuals (33.6% with NAFLD diagnosed by ultrasound).^7^ In a univariate analysis, NAFLD was associated with an increased risk of cancer (HR 1.32). Allen et al., investigating a cohort of 4722 patients with NAFLD and 14 441 matched controls, found an increased risk of cancer (incidence rate ratio 1.9). Wang et al., in a cohort of 54 187 men (32% with NAFLD diagnosed by ultrasound), reported an increased risk of cancer in patients with NAFLD (HR 1.09 in univariate analysis).^13^ Using population‐based registries with a high validity for the outcomes studied, our results corroborate the above findings.

The association between NAFLD and the risk of colorectal cancer has been investigated in several studies. Most of these studies included Asian populations.^14^, ^15^, ^26^, ^27^, ^28^, ^29^, ^30^, ^31^ In a meta‐analysis of 11 studies, Mantovani et al. reported an association between NAFLD and risk of colorectal cancer.^15^ The included studies used different diagnostic modalities for NAFLD and heterogeneity between studies was rather large.^15^ In the above‐mentioned study by Kim et al., NAFLD was associated with an increased risk of colorectal cancer in men (HR 2.21) but not in women,^7^ a finding in line with our results. In a more recent meta‐analysis from 2021 by Mantovani et al., including results from eight studies including over 44 000 patients with NAFLD, an association between NAFLD and risk of colorectal cancer was also reported (HR 1.64). The included studies had significant heterogeneity (*I* ^2^ = 57.9%) and all but one study were on Asian cohorts.^12^ Contrary to our study, the 2021 study by Mantovani et al. reported associations between NAFLD and risk of oesophageal (HR 1.93), stomach (HR 1.81), lung (HR 1.30) and breast cancer (HR 1.39).^12^ The different ethnic constitution of our cohorts and the larger population size in the study by Mantovani et al. could explain these differences.

In a meta‐analysis, Liu et al. investigated the association between NAFLD and the risk of cholangiocarcinoma, colon, breast, gastric, pancreatic, prostate and oesophageal cancer.^32^ In logistic regression, NAFLD was associated with colorectal (odds ratio [OR] 1.72 [*p* < .01]), gastric (OR 1.74 [*p* = .01]) and prostate cancer (OR 1.36 [*p* < .01]).^32^ The heterogeneity between the studies investigating colorectal, gastric and prostate cancers was significant (*I* ^2^ 83.5% for colorectal, 73.6% for gastric and 81.9% for prostate cancer).

Kanwal et al. reported an increased risk of HCC (aHR 7.62 compared to patients without NAFLD) in 296 707 patients with NAFLD (diagnosed using consecutively increased alanine aminotransferase levels). These NAFLD patients had received healthcare from the Veterans Health Administration.^10^ An IR of 0.08 cases of HCC per 1000 person‐years in patients with NAFLD without cirrhosis was reported. In comparison, we found an IR of 0.7 cases of NAFLD per 1000 person‐years in NAFLD patients without cirrhosis (Table S2). The difference in diagnostic methods and the inclusion of study participants from a population‐based registry of patients receiving specialized care in our study could explain these differences.^10^

Simon et al. reported that, after adjusting for several important confounders, patients with biopsy‐proven NAFLD had an increased risk of cancer compared to matched controls (aHR 1.27, 95% CI = 1.18–1.36).^33^ This finding is supported by our study using a less selected cohort with study participants who were identified and included using ICD codes. Since the study by Simon et al. included all Swedish patients with biopsy proven from 1966 to 2016, our two cohorts likely included many of the same patients. As in our cohort, the strongest association was observed between NAFLD and the risk of HCC development (aHR 17.08, 95% CI = 11.56–25.25). In contrast to the findings of our study, Simon et al. did not report an association between NAFLD and risk of colorectal and uterus cancer, which could be explained by the different selection criteria of our cohorts.^33^

Our study has several strengths. First, we used data from high‐quality population‐based registries to ascertain both exposure and outcomes. This approach reduces selection bias and increases the generalization of the results to countries with a similar population to Sweden. Second, we compared the risk of cancer in patients with NAFLD to the risk in the general population. Third, owing to the high validity of malignancies in the SCR, we likely had a high capture rate for the assessed outcomes.

The study had some limitations. In general, these are related to the register‐based design, which introduces the possibility of selection bias and limited possibility to identify confounders. First, because NAFLD is generally underdiagnosed and we used ICD codes from hospital discharges (and from specialized care outpatients visits since 2001), we likely underestimated the prevalence of NAFLD in the study population, leading to false low‐risk estimates in our study. Second, the NPR does not include patients from primary care, which may introduce a selection bias towards more severe cases with NAFLD. However, the relatively low prevalence of cirrhosis in our cohort suggests that selection bias is not a concern. However, this low prevalence of cirrhosis could also be attributed to misclassification bias as a result of suboptimal coding. To assess this possibility, we investigated how many of the 40 NAFLD patients without cirrhosis at baseline but who received a diagnosis of HCC during follow‐up had been diagnosed with cirrhosis after baseline but before their HCC diagnosis. Excluding these individuals (*n* = 10) from the Cox regression analysis of the risk of HCC, we observed similar results to those of the main analysis. It is possible that some patients diagnosed with NAFLD in our cohort consumed excess amounts of alcohol and had undiagnosed alcohol‐related liver disease. Therefore, we excluded all individuals with a diagnosis of alcohol or drug abuse. Patients with a formal diagnosis of NAFLD might not be representative of patients with NAFLD in the general population. The risk of cancer might be higher in patients with a formal diagnosis, if these are in general sicker. Also, underestimation of the possible confounders of interest in both patients with NAFLD and their general population controls might occur in this study, possibly more so in controls since they are not followed actively in the used registers. Hence, the risk estimates for cancer obtained in this study might be falsely high. However, this do not in a meaningful manner affect our conclusions that risk of extrahepatic cancer in NAFLD is in general low. It is also possible that some patients diagnosed after baseline had already developed cancer before baseline. Thus, we introduced a lag time value in which we only considered outcomes occurring at least 1 year after baseline. The baseline covariates included in the regression models (i.e. diabetes, COPD, hypertension and hyperlipidaemia) were ascertained through the NPR. Because patients with NAFLD could be more likely to contact healthcare than individuals without NAFLD, patients with NAFLD could also be more likely to receive a diagnosis of one or more of the covariates. Finally, we relied on COPD as a proxy for smoking. Such a proxy, however, only captures the most severe cases. Thus, some residual confounding from smoking in the estimates is possible.

Future studies should include cohorts with a more precise definition of NAFLD, including a higher level of granularity of liver fibrosis stage, have a longer follow‐up and more detailed data on important confounding comorbidities and lifestyle‐related risk factors. In general, there is always risk of bias in register‐based, observational studies, and therefore the results should be interpreted with some caution. Verifying the results from this study with other studies is important.

In summary, using a population‐based cohort of patients with NAFLD compared to controls without NAFLD, we found NAFLD to be associated with a slightly increased risk of cancer (primarily HCC). In general, the risk of cancer was low and these results do not call for specific surveillance of cancer in patients with NAFLD.

## CONFLICT OF INTEREST

None.

## Funding information

HH received research grants from Region Stockholm (postdoctoral appointment), the Ruth and Richard Julin Foundation, Tore Nilssons Foundation for Medical Research, the Åke Wiberg Foundation and the Swedish Society of Medicine.

## Supporting information


eTable 1

Tables S1–S2

## Data Availability

Due to the nature of this research, participants of this study did not agree for their data to be shared publicly, so supporting data is not available.
