# Supplementary material for: Risk of hepatic and extrahepatic cancer in NAFLD: A population‐based cohort study
Source: Liver Int. 2022 Feb 24;42(4):820–8. doi: 10.1111/liv.15195 (PMC9306866; doi:10.1111/liv.15195)
Supplement: Supplementary file 1 — eTable 1 [file LIV-42-820-s002.docx]

| **eTable 1. ICD-codes for all diagnoses used in the study** | | | |
| --- | --- | --- | --- |
|  | **ICD-7**  **(cancer registry)** | **ICD-9** | **ICD-10** |
| **Exposure** |  |  |  |
| NAFLD | - | 571W | K75.8 K76.0 |
| **Primary outcome** |  |  |  |
| Any type of cancer (except non-melanoma skin cancer) | 140 - 207, except 191 | - | - |
| **Secondary outcomes** |  |  |  |
| Hepatocellular carcinoma | 155.0 | - | - |
| Colorectal cancer | 153 154 | - | - |
| Stomach cancer | 151 | - | - |
| Kidney cancer | 180 | - | - |
| Bladder cancer | 181.0 | - | - |
| Cervix cancer | 171 | - | - |
| Ovarian cancer | 175.0 | - | - |
| Uterine cancer | 172 173 174 | - | - |
| Breast cancer | 170 | - | - |
| Lung cancer | 162 | - | - |
| Esophageal cancer | 150 | - | - |
| Prostate cancer | 177 | - | - |
| **Exclusion criteria** |  |  |  |
| Alpha-1-antitrypsine deficiency | - | 277G | E88.0A E88.0B |
| Alcoholic liver disease | - | 571A 571B 571C 571D | K70 |
| Autoimmune hepatitis | - | - | K75.4 |
| Budd-Chiari syndrome | - | 453A | I82.0 K76.5 |
| Hemochromatosis | - | 275A | E83.1 |
| PBC | - | 571G | K74.3 K74.5 |
| PSC | - | (555 or 556) + 576B | (K50 or K51) + K83.0 |
| Wilson's disease | - | 275B | E83.0B |
| Viral hepatitis | - | 070 571E | B15 B16 B17 B18 B19 |
| Portal vein thrombosis | - | - | I81.9 K75.1 |
| Alcohol or drug abuse | - | 291A 291B 291C 291D 291E 291W 291X 292 303 304 305 359E 425F 535D 655F | E24.4 F10-F19 G31.2 G62.1 G72.1 I42.6 K29.2 K85.2 K86.0 O35.4 X65 Y15 Y91 |
| Liver transplants | - | V42H 5200 | Z94.4 JJC00 JJC10 JJC20 DJ005 DJ006 JJC30 JJC40 |
| **Comorbidities** |  |  |  |
| Cirrhosis | - | 571.5 | K74.6 |
| Diabetes | - | 250 | E10 - E14 |
| COPD | - | 491 492 496 | J41 - J44 |
| Hypertension | - | 401 402 403 404 405 | I10 I11 I12 I13 I15 |
| Hyperlipidemia | - | 272 | E78 |

**eTable 1.** Abbreviations; PBC=Primary biliary cholangitis, Primary sclerosing cholangitis, COPD=Chronic obstructive pulmonary disease.
