# Supplementary material for: Risk of hepatic and extrahepatic cancer in NAFLD: A population‐based cohort study
Source: Liver Int. 2022 Feb 24;42(4):820–8. doi: 10.1111/liv.15195 (PMC9306866; doi:10.1111/liv.15195)
Supplement: Supplementary file 2 — Tables S1–S2 [file LIV-42-820-s001.docx]

| Supplementary table 1. Risk of all cancers and specific cancer types in persons with NAFLD without cirrhosis at or before baseline and matched reference individuals | | | | | | |
| --- | --- | --- | --- | --- | --- | --- |
| Type of cancer | Incident cases | | Incidence rate (95% CI) per 1000 PY | | HR (95% CI) | aHR (95% CI) |
|  | NAFLD, n=8232 (n, %) | Reference individuals, n=69514 (n, %) | NAFLD | Reference individuals |  |  |
| All cancers | 518 (6.3) | 4616 (6.6) | 9.6 (8.8-10.5) | 8.6 (8.3-8.8) | 1.2 (1.1-1.3) (p<0.01) | 1.2 (1.1-1.3) (p<0.01) |
| Hepatocellular carcinoma | 40 (0.5) | 34 (0.0) | 0.7 (0.5-1.0) | 0.1 (0.0-0.1) | 13.3 (8.5-21.0) (p<0.01) | 10.8 (6.3-18.6) (p<0.01) |
| Colon and rectum | 77 (0.9) | 635 (0.9) | 1.4 (1.1-1.7) | 1.1 (1.0-1.2) | 1.4 (1.1-1.7) (p<0.01) | 1.4 (1.1-1.8) (p<0.01) |
| Stomach | 10 (0.1) | 75 (0.1) | 0.2 (0.1-0.3) | 0.1 (0.1-0.2) | 1.5 (0.8-2.9) (p=0.19) | 1.3 (0.7-2.7) (p=0.41) |
| Kidney | 21 (0.3) | 108 (0.2) | 0.4 (0.2-0.6) | 0.2 (0.2-0.2) | 2.3 (1.5-3.5) (p<0.01) | 2.1 (1.3-3.3) (p<0.01) |
| Bladder | 37 (0.4) | 194 (0.3) | 0.7 (0.5-0.9) | 0.3 (0.3-0.4) | 2.4 (1.7-3.3) (p<0.01) | 2.5 (1.8-3.5) (p<0.01) |
| Cervix | 12 (0.1) | 147 (0.2) | 0.2 (0.1-0.4) | 0.3 (0.2-0.3) | 0.8 (0.5-1.4) (p=0.45) | 1.0 (0.6-1.7) (p=0.94) |
| Ovary | 8 (0.1) | 84 (0.1) | 0.1 (0.1-0.3) | 0.2 (0.1-0.2) | 1.0 (0.5-3.0) (p=0.96) | 0.9 (0.4-2.0) (p=0.86) |
| Uterus | 25 (0.3) | 132 (0.2) | 0.4 (0.3-0.7) | 0.2 (0.2-0.3) | 1.8 (1.2-2.7) (p<0.01) | 1.8 (1.2-2.7) (p<0.01) |
| Breast | 68 (0.8) | 611 (0.9) | 1.2 (1.0-1.6) | 1.1 (1.0-1.2) | 1.1 (0.9-1.4) (p=0.29) | 1.1 (0.9-1.5) (p=0.29) |
| Lung | 36 (0.4) | 378 (0.5) | 0.6 (0.5-0.9) | 0.7 (0.6-0.7) | 1.0 (0.7-1.4) (p=0.93) | 1.0 (0.7-1.4) (p=0.96) |
| Esophagus | 2 (0.0) | 42 (0.1) | 0.0 (0.0-0.1) | 0.1 (0.1-0.1) | 0.4 (0.1-1.8) (p=0.26) | 0.5 (0.1-2.2) (p=0.38) |
| Prostate | 74 (0.9) | 1002 (1.4) | 1.3 (1.1-1.7) | 1.8 (1.7-1.9) | 0.8 (0.7-1.0) (p=0.09) | 0.9 (0.7-1.1) (p=0.33) |

**Supplementary table 1.** Total number of cancer diagnoses, incidence rates per 1000 person-years and crude and adjusted hazard ratios for incident cancers in persons with a diagnosis of NAFLD without cirrhosis in Sweden between 1987-2016 compared to age, sex and living location matched reference individuals without cirrhosis.

aHR = adjusted for diabetes, hypertension, hyperlipidemia and chronic obstructive pulmonary disease.

Abbreviations; aHR=adjusted hazard ratio.

| Supplementary Table 2. Competing risks regression for all cancer and death from all cancer, and HCC and death from HCC.. | | |
| --- | --- | --- |
|  | SHR (95% CI) | aSHR |
| All cancer | 1.22 (1.13-1.32) (p<0.01) | 1.10 (1.02-1.20) (p=0.02) |
| HCC | 11.43 (8.26-15.82) (p<0.01) | 8.17 (5.68-11.75) (p<0.01) |

**Supplementary Table 2**. Competing risks regression for all cancer and death from all cancer, and HCC and death from HCC, with death of other causes acting as competing risk. aSHR = adjusted for diabetes, hypertension, hyperlipidemia and chronic obstructive pulmonary disease.

aSHR=adjusted subdistribution hazard ratio.
